# High-stress study of bioinspired multifunctional PEDOT:PSS/nanoclay nanocomposites using AFM, SEM and numerical simulation

**DOI:** 10.3762/bjnano.8.207

**Published:** 2017-10-04

**Authors:** Alfredo J Diaz, Hanaul Noh, Tobias Meier, Santiago D Solares

**Affiliations:** 1 Department of Mechanical and Aerospace Engineering, The George Washington University, Washington, DC 20052, United States

**Keywords:** biomimetics, conductive AFM, conductive nanocomposites, contact-resonance force microscopy, multifrequency AFM, transparent coatings

## Abstract

Bioinspired design has been central in the development of hierarchical nanocomposites. Particularly, the nacre-mimetic brick-and-mortar structure has shown excellent mechanical properties, as well as gas-barrier properties and optical transparency. Along with these intrinsic properties, the layered structure has also been utilized in sensing devices. Here we extend the multifunctionality of nacre-mimetics by designing an optically transparent and electron conductive coating based on PEDOT:PSS and nanoclays Laponite RD and Cloisite Na^+^. We carry out extensive characterization of the nanocomposite using transmittance spectra (transparency), conductive atomic force microscopy (conductivity), contact-resonance force microscopy (mechanical properties), and SEM combined with a variety of stress-strain AFM experiments and AFM numerical simulations (internal structure). We further study the nanoclay’s response to the application of pressure with multifrequency AFM and conductive AFM, whereby increases and decreases in conductivity can occur for the Laponite RD composites. We offer a possible mechanism to explain the changes in conductivity by modeling the coating as a 1-dimensional multibarrier potential for electron transport, and show that conductivity can change when the separation between the barriers changes under the application of pressure, and that the direction of the change depends on the energy of the electrons. We did not observe changes in conductivity under the application of pressure with AFM for the Cloisite Na^+^ nanocomposite, which has a large platelet size compared with the AFM probe diameter. No pressure-induced changes in conductivity were observed in the clay-free polymer either.

## Introduction

Bioinspired material designs have been at the forefront of artificial nanocomposite developments [1]. Complex architectures in bones [2–3], mollusk shells [4–5], and fish scales [6–7], among other biological systems, offer enhancements over the intrinsic properties of the individual constituents. Particularly, nacre has received much attention due to its remarkable mechanical properties and convoluted structure. Nacre is composed of 95% aragonite platelets separated by 5% organic matter, displaying a multiscale hierarchical structure with features ranging from molecular to macroscopic scales [8]. The experimental approach to produce artificial nacre is usually based on a self-assembly process involving clay nanosheets (nanoclays) and polymers. In general, the dispersed polymer-coated nanoclays naturally organize by liquid removal, leading to an organic-inorganic multi-layered assembly, the so-called brick-and-mortar structure [9–10].

Although the mechanical properties of the nanoclay/polymer nanocomposites have not matched those of nacre [8], other interesting properties have been reported besides being lightweight and potentially transparent [11]. Gas permeability experiments have shown that the brick-and-mortar structure significantly reduces diffusion of gases across it [12–14]. The suggested mechanism for the decreased permeability is an increased-tortuosity path for the gas, due to the presence of the nanoclay particles [14–15]. Since the nanoclay prevents oxygen from diffusing into the organic phase, it also results in a fire-retardant material [16–17]. In recent years the focus has also shifted towards extending the functionality of nanocomposites to the design of stimuli-responsive systems [18]. In nanoclay-based systems, external stimuli reshape the organic phase conformation, and as a result, a measurable change in optical (or other) properties is obtained [19]. Zhuk et al. [20] designed a temperature, pH and salt concentration sensing system based on poly(*N*-isopropylacrylamide) (PNIPAM) and nanoclays, to operate in an aqueous environment. Similar layered structures have shown suitability for sensing humidity [21] and for temperature-dependent switching of electrochemical behavior using nanoclay substitutes [22].

Recently, nacre-mimetic electrically conductive nanocomposites have been reported, one of which exhibited good electrical conductivity and excellent mechanical properties [23] and the other of which demonstrated feasibility as an electrode [24]. The multifunctionality of the brick-and-mortar structure is an important advantage compared to other architectures, for electrically conductive nanocomposites. For example, a recently developed piezoelectric sensor exploited the PVDF/nanoclay structure in electrospun fibers for sensing a voltage response to loading while maintaining good flexibility within an application as smart clothing [25]. The integration of hard particles into a soft matrix has also gained interest for the design of advanced functional materials [25–27]. Recent studies have investigated the relationship between applied stress and movement of the embedded hard particles [26]. When periodicity of inclusions is obtained in the nanocomposite, tunability of the photonic [27] or phononic [28] band gap has been achieved by applying mechanical stress.

In this paper, we leverage the brick-and-mortar structure and properties, in combination with a variety of atomic force microscopy (AFM) methods, to investigate the high-pressure response of a bioinspired transparent and electrically conductive nanocomposite. Specifically, a transparent PEDOT:PSS/nanoclay coating is fabricated by a simple solvent casting method. Nanoscale out-of-plane current is studied with conductive-AFM (C-AFM) and correlated with the film mechanical parameters obtained from contact-resonance force microscopy (CRFM). Then, high-pressure (few GPa) is applied locally to the surface by means of bimodal AFM, which is expected to modify the spacing of the embedded nanoclay particles, causing a change in the electrical conductivity and local mechanical properties. Bimodal AFM, together with numerical AFM simulation and SEM, is used to infer the internal distribution of the nanoclay. Finally, a one-dimensional multibarrier potential is used to explain the changes in electrical conductivity across the coating under the application of pressure. The study is carried out for two different nanoclays, namely Laponite RD and Cloisite Na^+^.

## Results and Discussion

### Transparency and electromechanical properties

PEDOT:PSS is a highly conductive polymer that can be designed to have high optical transparency [29]. In “thick” PEDOT:PSS films, the blue color of PEDOT dominates the optical properties [30]. Thickness reduction is customary in order to achieve highly transparent films. Conductive and transparent interfaces have received special attention as an integral component in organic photovoltaics and organic light-emitting diode systems. Developing a transparent pressure-responsive nanocomposite has potential application within touchscreen displays. To synthesize a transparent nanocomposite coating, the approach is to dilute the “as prepared” core/shell dispersion. The amount of solids available per unit volume decreases, resulting in thinner films when the solution is casted. Four different dilutions (1:2, 1:4, 1:8 and 1:16) were used for casting samples in the present study, and the coating was characterized using optical transmittance spectroscopy. Nanocomposites prepared from Laponite RD (LAP) and Cloisite Na^+^ (montmorillonite, MTM) were also compared to the bare PEDOT:PSS to evaluate the change in transmittance caused by the addition of nanoclay.

**Figure 1:**
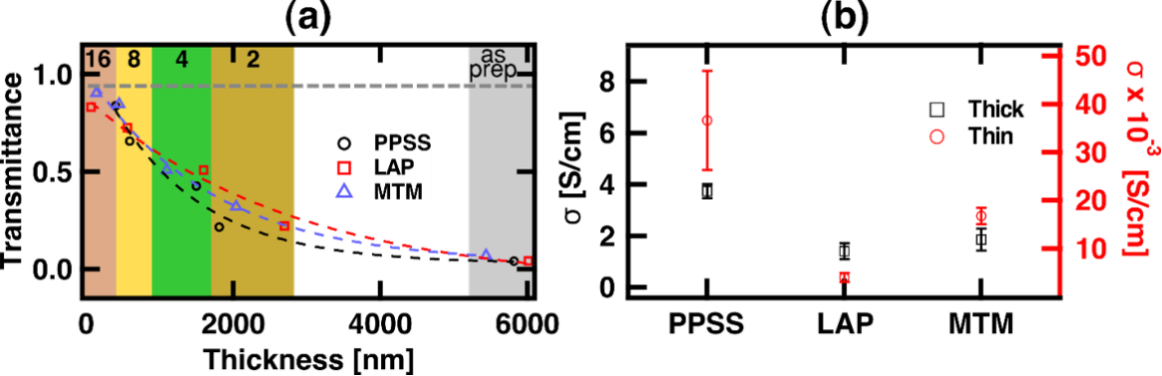
(a) Summary of optical characterization. The transmittance for the different samples is plotted versus the thickness for a wavelength λ = 550 nm. The colored areas correspond to the different dilution factors, which are specified at the top of the figure. The dashed grey line shows the transmittance of the substrate (glass slide). All the results are fitted to exponential functions, shown in dashed lines. (b) Electrical conductivity summary showing the average and one standard deviation range, acquired using C-AFM. The results for the thick and thin samples are plotted on different vertical axes.

Figure 1a demonstrates the tunability of the nanocomposites’ transparency by changing the thickness. The samples are casted onto glass substrates and the grey dashed line represents the transmittance across the bare substrate. The trend shows that, as the thickness decreases, more light is transmitted, i.e., the transmittance approaches the value of the bare glass slide, as expected. The samples casted from the 1:16 dilution are referred to as the thin and transparent coatings, since that dilution ratio produced the most transparent and thinnest sample. It is noteworthy that the addition of the nanoclay did not significantly affect the transmittance in our experiments. To compare the decay in transmittance, the curves are fitted to a decaying exponential function, using the least squares method. The exponential decay constants are: 0.00081/nm for PPSS, 0.00036/nm for LAP and 0.0006/nm for MTM, which means that the transmittance decays faster in PEDOT:PSS with respect to thickness, compared to the LAP nanocomposite, and the MTM nanocomposite falls in between the two. In both cases, the addition of the nanoclay reduces the decay constants. Also, it is known that Laponite RD-based nanocomposites produce films with higher transparency compared to Cloisite Na^+^ and that irregularities must be prevented to reduce detrimental light scattering within the film [11]. It has also been previously shown that the addition of nanoclay helps in the formation of ordered phases in the polymer [25,31], which may lead in PEDOT:PSS nanocomposites to morphologies that favor the transmission of light at higher thicknesses. Nevertheless, the optical transparency of the nanocomposite is still governed primarily by the polymer, PEDOT:PSS. Analyzing the full spectra (Supporting Information File 1, Figure S1a–c), one can see that the transmission is maximum on the blue end of the spectrum and decreases when moving into the red, which is the typical behavior for PEDOT:PSS films [30].

The most relevant feature of PEDOT:PSS is its high electrical conductivity. Localized electrical properties of nanocomposites have been widely studied using C-AFM [32–33]. In C-AFM [34], a conductive nanoscale probe is scanned in continuous contact (static mode) with the sample while obtaining the topography. The local current is mapped by applying a bias voltage. The electrical properties of the nanocomposites were investigated using C-AFM and compared to the bare polymer. In C-AFM, the applied voltage is defined by the user depending on the material. The mechanisms of current transport through organic media have been under investigation for many years [35] and it is known that there are three distinct regions in the current vs voltage (*I*–*V*) curves for organic conductors [35–37]. Only at low voltages, transport follows a linear, ohmic behavior [36]. From moderate to high voltages, non-linear behavior is expected and is controlled by distributed carrier traps within the organic layer. In order to use Ohm’s law to analyze the data, the measurements should be performed within the linear regime. Due to the difficulty and unreliability of holding the AFM probe steady during measurement (due to high drift in ambient conditions), which would be required in order to maintain a constant contact area during a current-voltage (*I*–*V*) measurement, we confirm linearity of the *I*–*V* behavior by acquiring C-AFM images in which the voltage bias polarity is changed in the middle of the image during scanning, as in Supporting Information File 1, Figure S2. The conductive spots switch from −5 nA (top of the image, blue spots) to +5 nA (bottom of the image, yellow spots) when the polarity is changed from 100 to −100 mV in the middle of the image, while maintaining a background of zero current (green). Given the linearity observed for this voltage range, all samples were imaged with 100 mV of bias voltage.

The electrical conductivity of the various samples was analyzed using the expression σ = *IL*/*VA* (based on the definition of electrical resistivity and Ohm’s law), where *I* is the total current (sum of all pixels), *L* is the thickness of the film, *V* is the applied bias voltage and *A* is the scanned area (image size). The results are summarized in Figure 1b. Each point represents the average of 6 full images and their combined standard deviation. Since many experiments were performed, a random order was used, alternating thick and thin samples. Specifically, a random sample (either thick or thin) was placed in the microscope and one image was taken. Afterwards the sample was changed, and the process was repeated 18 times (6 times in total per sample).

From Figure 1b it is observed that the conductivity for the thick samples is inside the range given in the literature for PEDOT:PSS [29]. Additionally, the average conductivity trend for the three samples (PPSS, LAP and MTM) is similar for both cases (thick and thin). Comparing the thick and thin samples, one can observe that the calculated conductivity varies several orders of magnitude. The calculation of conductivity depends directly on the coating thickness, which ranges from ≈6 µm in the thick samples to ≈100 nm in the thin samples (Figure 1a). It is also known that solvent casting parameters affect the morphology of polymers [38] and that the morphology highly influences the conductivity [39], although this was not specifically measured in the present study.

For the thick PEDOT:PSS/nanoclay nanocomposite films, it has been shown that thermal treatments can increase the macroscopically measured conductivity to values near those of bare PEDOT:PSS [23]. In this case, no thermal treatments were performed. The thin samples showed a decrease in conductivity possibly associated with non-favorable structural conformations. It has been reported that the percolation of current across a PEDOT:PSS film increases as the thickness increases, since it is more probable to achieve larger PEDOT agglomerates [40]. When multiple PEDOT grains are stacked, the number of interconnections increases and the long-range connectivity increases, thus leading to improved electrical conductivity. For an electric field applied perpendicular to the film, the conductivity of PEDOT:PSS has been shown to be approximately three orders of magnitude lower when compared to the in-plane case, for thin films [41]. The supposed reason for this is the existence of a quasi-continuous 1–2 nm layer of insulative PSS separating PEDOT-rich particles in the perpendicular direction [41].

Another important observation is that the addition of the nanoclay to PEDOT:PSS does not significantly decrease the conductivity (the values remain in the same order of magnitude), as can be seen when comparing LAP and MTM with PPSS in Figure 1b. The structure of the nanoclays used, Laponite RD [42] and Cloisite Na^+^ [43], consists of negatively charged platelets separated by sodium cations. The stacks separate, dispersing negatively charged non-polar platelets and Na^+^ ions, when exfoliated in deionized water. Several investigations have shown that solvent processing of PEDOT:PSS significantly increases the electrical conductivity [30,44–45]. In general, the mechanism behind the increased conductivity is associated with a morphological change caused by the treatment. PEDOT:PSS is a polymer blend in which PEDOT is conductive, hydrophobic and positively charged, and PSS is hydrophilic and charged negatively. The post-treatments take advantage of the polymers’ properties to modify the structure. When ions from concentrated sulfuric acid [45] or methanol (hydrophilic [44]) are added, PEDOT:PSS undergoes a structure rearrangement that results in a morphology that favors electron conduction. The nanoclay dispersions have the potential to modify the morphology of the polymer by secondary interactions due to the nanoclay charge, sodium cations and hydrophobicity, thus influencing the conductivity.

The mechanical properties of the samples (PPSS, LAP and MTM) were compared using CRFM. In CRFM, the resonance frequency and quality factor of the mechanically-coupled tip–sample surface system are measured [46–48]. The contact-resonance frequency, which is higher than the free cantilever resonance frequency, is directly related to stiffness (larger stiffness leads to larger frequency and vice-versa) [49], while the quality factor maps the sample damping of the cantilever tip oscillation (greater dissipation leads to lower quality factor and vice-versa) [50]. The contact-resonance frequency and quality factor are often referred to as mechanical parameters. Although, there are methods to approximately calibrate for the Young’s modulus, they require a standard reference sample with similar properties to the unknown sample, and this includes the surface properties [51]. Additionally, polymer mechanical properties are very sensitive to the frequency of the measurement due to their viscoelasticity (rate-dependent behavior) [52]. In CRFM, an additional challenge is the inability to probe at frequencies lower than the kilohertz to megahertz range because of the resonance properties of typical cantilevers (traditional testing of polymeric or biological materials is performed at a frequency range several orders of magnitude lower [53]). For these reasons, CRFM typically only provides mapping of the relative surface properties, although with high spatial resolution. Given the complexity in the interpretation of the measurements as quantitative mechanical properties, the mechanical parameters (contact-resonance frequency and quality factor) of the fabricated samples are used in this study, whereby the same cantilever and imaging conditions are used for all samples, which enables discussion of their relative properties and changes.

**Figure 2:**
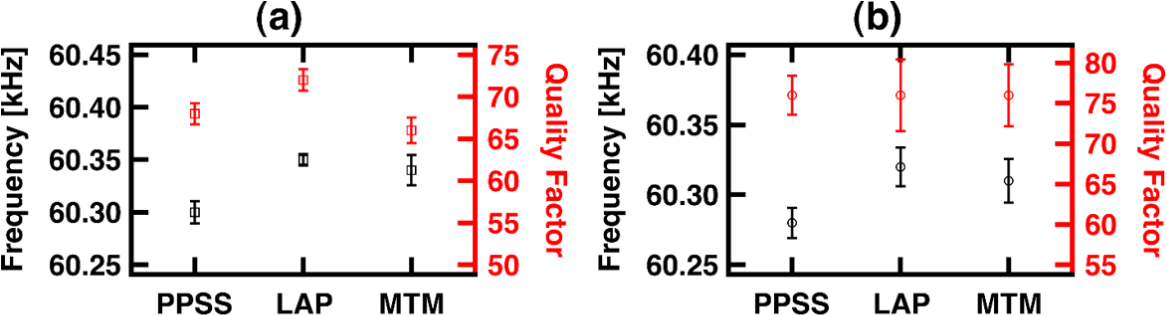
Summary of mechanical parameters for all samples, obtained using CRFM: (a) thick and (b) thin samples. Average frequency and quality factor values were obtained by fitting a Gaussian distribution to a histogram of the images. The one-standard-deviation range is also shown.

Figure 2 shows a summary of the mechanical parameters for thick and thin samples using the same cantilever and characterizing the samples in a random sequence. It is known, from macroscopic tensile testing, that the addition of nanoclays increases the stiffness of polymers [54]. As expected, for both cases (thick and thin) the average measured contact-resonance frequency for the nanocomposites is higher than the frequency for PPSS, which means that the addition of the nanoclay results in a stiffer coating.

Comparing thick and thin samples, it is observed that the average frequency is lower for the thin samples, but the trends are similar. The reduction of stiffness for the thin samples comes with a reduction in the conductivity (Figure 1b), which suggests that there is a different morphological arrangement in the thin samples. Since all the thin samples showed a reduction in the electromechanical response, we conclude that the morphology is ruled by the conductive polymeric matrix, PEDOT:PSS. It is known that the electrical conductivity of PEDOT:PSS films decreases as the thickness decreases because of the resulting unfavorable structure [40]. Despite the extensive use of PEDOT:PSS, only few mechanical property investigations have been performed [40], which have mostly dealt with microscale film thicknesses [55–56]. Films with nanoscale thickness have shown lower Young’s modulus, *E*, compared to thicker reported values [40]. The decrease in *E* observed for the thin films was associated with the intrinsic structure of the nanofilm, in which only a loose packing of PEDOT-rich grains can be obtained. In thicker films, the interconnections between neighbor grains are improved by the stacked, ‘‘pancake-like’’ structure, which in turn improves the mechanical properties [57–58]. The results from C-AFM and the contact-resonance frequency of CRFM confirm the change in packing of the thin coatings by measuring an inferior electro-mechanical performance compared to the thick films. It is noteworthy that most applications of PEDOT:PSS require thicknesses of tens of nanometers, for example in polymer solar cells [59].

It is also worth noting that in both cases, thick and thin, there was a decrease in the average contact-resonance frequency for MTM compared to LAP. In macroscale tension testing, MTM has shown higher stiffness than LAP [11]. CRFM measures the surface and the results are interpreted using a 1-dimensional analysis that neglects in-plane surface elastic forces or three dimensional tip artifacts [51], but has proven to be a valuable technique for semi-quantitative comparisons [50,60]. It is also known that nanoclays with larger size produce a skin layer [11], which is a heterogeneous surface layer that forms during the evaporation of the dispersion. Given the size difference of the nanoclays used, this layer is expected to have a more appreciable effect in MTM than in LAP. With regards to stiffness, the MTM nanocomposite showed an unexpected behavior given its platelet size. One would expect the larger platelet size to lead to a higher stiffness, but instead MTM showed lower stiffness compared to LAP. These incongruences can be explained in terms of the skin layer formation, which in MTM is more heterogeneous and less organized than in LAP (hence also more loosely packed), as a result of the larger particle size. Recall also that the cantilever tip interacts with the outermost volume of the coating, which is the heterogeneous skin.

The average quality factor shows a different behavior compared to the average frequency. For the thick samples (Figure 2a), it follows the trend of the frequency for PPSS and LAP, but not for MTM. For the measured area, the quality factor for MTM is lower than for the other samples, translating into more damping. The damping comes either from the confined viscoelastic material (the polymer) trapped between the MTM platelets (they have higher surface area resulting in more interaction with the surrounding polymer) or from the more loosely-packed skin layer, resulting in more dissipation during oscillatory deformation. The thin samples (Figure 2b) showed very similar quality factors. As previously discussed, for thinner films the properties of the thin films are expected to be dominated by the morphology of PEDOT:PSS. In the case of the nanocomposites, the damping mechanisms are also dominated by the polymer (since the clays are comparatively incompressible), and hence the quality factor is very similar for all the thin samples.

**Figure 3:**
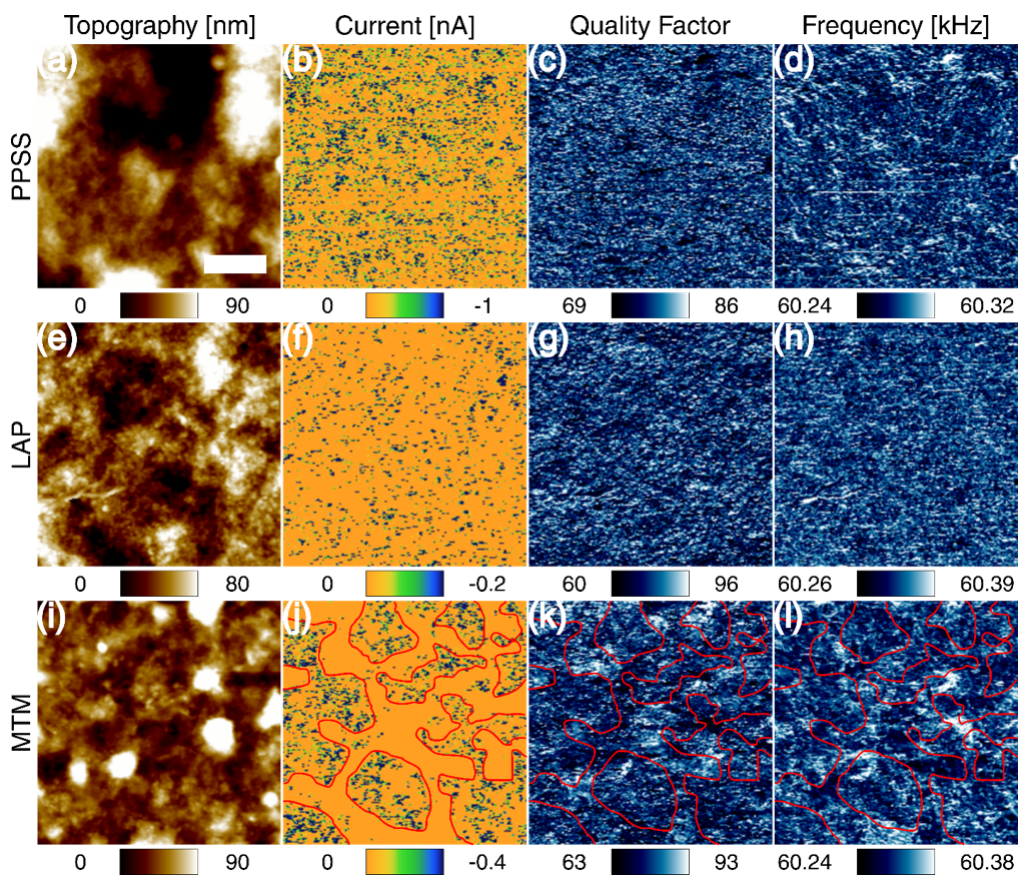
Typical correlated electro-mechanical properties of the thin samples: PEDOT:PSS (a–d), Laponite RD nanocomposite (e–h) and Cloisite Na^+^ nanocomposite (i–l). The columns represent topography, current, quality factor and contact-resonance frequency, respectively. The red outlines relate the electrical conductivity and mechanical parameter behavior for MTM. The scale bar is 1 μm.

Consecutive operation of C-AFM and CRFM was used to investigate spatial correlations between the electrical and mechanical response of the nanocomposites. Since sequential imaging is subject to lateral drift (lateral movement in between images), an automatic cross-correlation was performed to eliminate the drift effects. Figure 3 shows the electro-mechanical response of the transparent samples. The out-of-plane current showed a different distribution for the three samples, especially for MTM. The MTM nanocomposite shows segregation in the current and mechanical parameters. The areas outlined in red show the relation of areas with no current to the mechanical parameters in the MTM sample. In this case, the areas with no current are related to areas with higher quality factor (lower dissipation) and frequency (stiffer), which contain the platelets. The PPSS and LAP samples showed a homogeneous distribution of the conductive spots across the surface. Thick samples showed a very similar response among the three systems (refer to Supporting Information File 1, Figure S3).

As already stated, the mechanical response of MTM is not as expected, since the mechanical properties measured at the surface suggest that it is less stiff than LAP. This contradicts the macroscale results and, as also stated above, suggests that skin formation plays a prominent role in the properties of the film, as measured with AFM. On the other hand, the addition of Laponite RD to PEDOT:PSS improves the mechanical properties without considerably affecting the electrical properties or the distribution of the conducting paths. For these reasons, the rest of this thin film investigation is focused on thin and transparent Laponite RD nanocomposites, which may have greater applicability as stiffer coatings, with the added multifunctionalities related to the nacre-like structure (i.e., gas barrier behavior, fire retardancy, etc.).

### Distribution of nanoclay in the film

**Figure 4:**
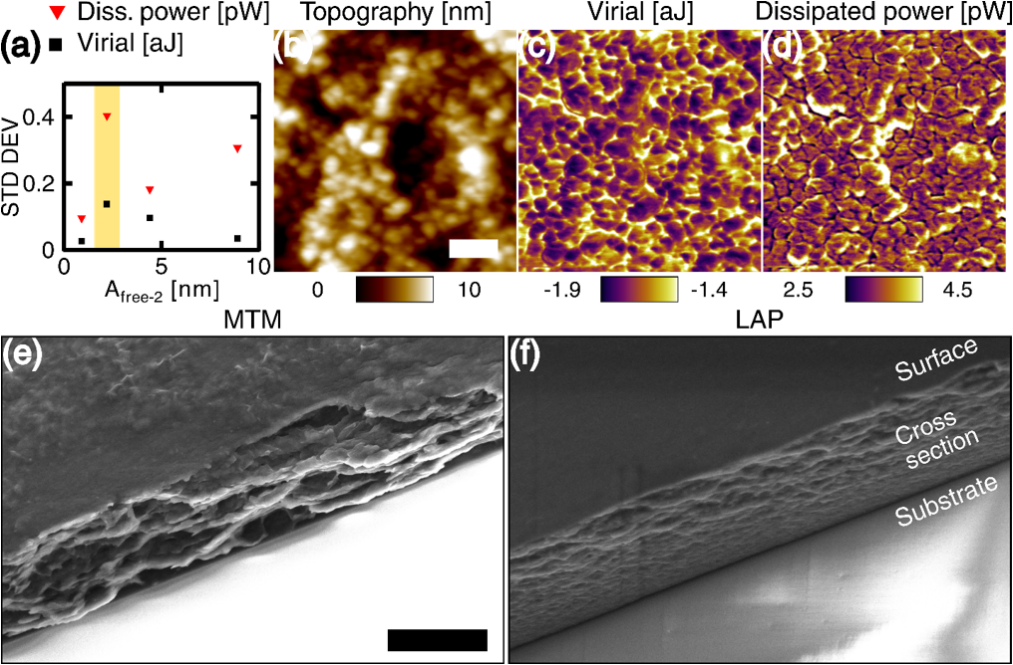
Measurement of virial and dissipated power for the second eigenmode of the cantilever in bimodal AFM, on the nanocomposite surface for 33/67 ratio of thin Laponite-RD/PEDOT:PSS. The first eigenmode was operated in repulsive regime (phase below 90°). The amplitude of the second eigenmode was varied in order to optimize the contrast in the energy quantities as described in [63] and summarized in (a), where the standard deviation of the measurement is plotted against the free oscillation amplitude. Images (b–d, topography, virial and dissipated power) correspond to the condition highlighted by the yellow area in (a). The scale bar (b) is 100 nm. (e–f) SEM characterization of the sample cross section. The scale bar in (e) is 5 μm.

The distribution of nanoclay near the surface was investigated by bimodal AFM for the Laponite RD films. Bimodal AFM [61] is a dynamic intermittent-contact AFM technique in which two eigenmodes of the cantilever are excited simultaneously, resulting in an enhanced material contrast and the ability to modulate indentation depth or applied force during imaging [62]. The strategy followed consisted of mapping the conservative and dissipative interactions between tip and sample, with high contrast, using the energy quantities virial and dissipated power [63]. Due to the difference in mechanical properties between the nanoclay particles and the polymer matrix, the distribution of the energy quantities readily reveals the position of the nanoclay platelets. During the acquisition of the images (Figure 4c,d) the contrast range (and consequently also the standard deviation) was maximized by varying the amplitude of the higher eigenmode to adjust contrast sensitivity. Both channels, virial and dissipated power, showed clear contrast, revealing the nanoclay particles. The virial showed dark domains that can be interpreted as areas with a greater amount of stored elastic energy during tapping, which correspond to the nanoclay. The dissipated power image shows dark boundaries, which originate from the confined polymer around the nanoclays. Analyzing the different regions measured in the energy channels with ImageJ, the measured average nanaoclay characteristic dimension was determined to be 24.1 nm in the virial image (210 particles) and 31.9 nm (169 particles) in the dissipated power image, compared to a 25 nm reference diameter of the Laponite-RD nanoclay. It is not surprising to see a difference between the two images, as dissipation and stored elastic energy contrast can be optimized independently to some extent and are thus not fully correlated with one another [63], and the dimensions observed in the image are also influenced by the dimensions and geometry of the tip. Nevertheless, the measured nanoclay dimensions are in both cases near the expected range (25 ± 4 nm [11]).

Composite materials based on nanoclays have a distinctive fractured surface morphology. One way of verifying the layered structure of the nanocomposites is to image the cross-section of the film after failure. The samples were broken by bending them, while submerged (frozen) in liquid nitrogen. Figure 4e–f shows the cross-sectional analysis with SEM for the LAP and MTM self-assembled nanocomposites. Typical fracture features for a nacre-mimetic composite are observed [11,64]. The Laponite nanoclay particles are smaller than the Cloisite particles [11], resulting in a straighter and cleaner fracture surface.

The analysis now shifts towards investigating the internal structure of the nanocomposite. There are several mechanical models that describe the behavior of nanocomposites, specifically focusing on nanoclay fillers. These models relate the mechanical response of the material, usually as a function of the concentration of nanoclay included in the system. Models have been developed for specific cases, for example for unidirectionally aligned particles and for particles with random orientation, among others. Since the calculation of the Young’s modulus with AFM methods is not reliable (as also discussed above) [51], the strain is measured instead and related to the peak imaging forces. The increased force obtained from bimodal AFM for increasing free oscillation amplitudes of the higher eigenmode [62] is used to compress the coating and measure the strain for different nanoclay concentrations. The predicted strain from different mechanical models was then compared to the experimental results.

**Figure 5:**
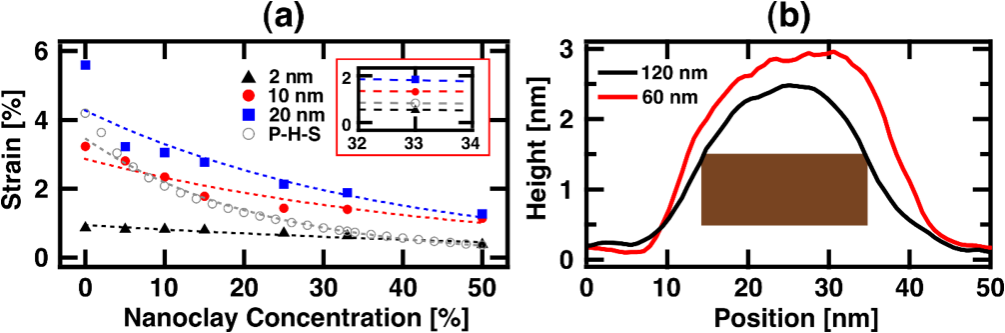
(a) Strain (vertical axis) produced by bimodal AFM in the nanocomposite film for different Laponite-RD nanoclay concentrations by weight (horizontal axis), using different values of the higher eigenmode free oscillation amplitude, indicated by the red, black and blue markers. The grey markers show the calculated strain using a combination of the modified Pukánszky and Hui–Shia model (P-H-S) [65–66]. All the results are fitted to exponential functions, shown in dashed lines. (b) Measurement of an individual Laponite-RD nanoclay platelet after polymer adsorption onto the surface.

The strain was calculated from bimodal AFM experiments. First, a reference measurement in repulsive-mode (phase below 90°) amplitude-modulation AFM was performed using the parameters given for the first eigenmode for Figure 5a in Supporting Information File 1. Then, the free amplitude of the higher eigenmode was gradually increased and the film thickness was calculated using the scratch method (see methods). The thickness obtained from AM-AFM (only one eigenmode) is used as the original thickness (*l*_0_) for the strain calculation, ε = (*l*_0_ − *l*)/*l*_0_, where *l* is the reduced thickness obtained from an increased amplitude of the second eigenmode. The results in Figure 5a show a significant decrease in strain as soon as the nanoclay is added to the polymer. The inset in Figure 5a shows that as the free amplitude of the higher eigenmode increases, the strain approaches 2% for 33% nanoclay concentration, which is the value used for all experiments. It is worth noting that the environmental humidity had a significant impact on the strain measurements. In fact, for samples that were stored and measured in very dry environments, no measurable deformation in the coating was obtained.

Assuming that the nanocomposite has a linear-elastic behavior, the strain can be estimated using models for composite materials’ Young’s modulus and stress behavior (σ = *E*ε). When the yield strength is considered as the limit of the linear response, the modified Pukánszky model [65] describes the stress. This model has been optimized for describing the yield strength of polymer/nanoclay composites and is based on the expression


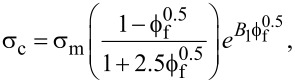


where c stands for ‘composite’, m for ‘matrix’, f for ‘filler’ (nanoclay), 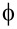 is the filler volume fraction and *B*_1_ is a parameter that depends on the interfacial interactions between the polymer and the nanoclay. The Hui–Shia model [66] describes the Young’s modulus of a polymer matrix filled with unidirectionally aligned nanoclay and has been tailored to represent the modulus in the direction perpendicular to the nanoclay surface. Also, this model considers the aspect ratio of the nanoclay, α = *t*/*D* (*t* is the thickness and *D* the diameter, where *t* = 1 nm and *D* = 25 nm for Laponite-RD). Several equations comprise this model:


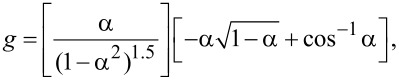



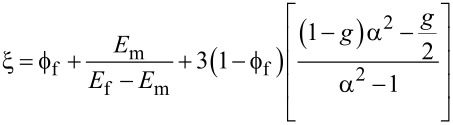


and


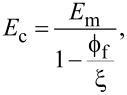


where *E* is the Young’s modulus. The volume fraction, 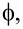 is related to the concentration by weight by


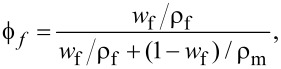


where *w* is the weight percent and ρ is the density [67].

Combining the above relations, the strain was calculated and plotted in Figure 5a (grey circles) using the parameters given in Supporting Information File 1, Table S1. The trend (grey dashed line) is an exponential decay, similar to the experimental results. The magnitude of the calculated strain lies between the experimental curves for 2 and 20 nm of free amplitude of the higher eigenmode (i.e., lowest and highest applied force). The models used were specifically developed for polymer/nanoclay composites with unidirectional alignment, thus the resemblance with the experimental results is consistent with the expectation that the coating contains ordered nanoclay. Supporting Information File 1, Figure S4 compares the behavior for strain using different models as a function of nanoclay concentration.

The thickness of individual nanoplatelets surrounded by polymer was also measured for two free amplitudes using amplitude-modulation AFM and is shown in Figure 5b. The measurement shows a thickness of approximately 3 nm (red curve) in lightly repulsive imaging mode (free amplitude of 60 nm). This sample was prepared by diluting the “as prepared” dispersion and depositing it onto a Si substrate in order to separate the individual nanoplatelets (the original dispersion ratio was 33/67 by weight of Laponite-RD and PEDOT:PSS, which was diluted with deionized water). The platelets are expected to be surrounded by polymer molecules due to the affinity they have for each other. To confirm the presence of polymer, the free amplitude was increased to 120 nm and the AFM measurement was repeated. This resulted in a reduction of the measured thickness, which is related to the compression of the polymer (since the nanoclay is highly incompressible). The images indicate that the 1 nm thick nanoclay has a ≈1 nm-thick coating of polymer on each face. Thus, when two nanoplatelets stack up, the expected interplatelet distance (d*) is ≈2 nm. As a reference, for a similar nanocomposite, Mäkiniemi et al. [23] have reported a d* of 2.08 nm, which was obtained from X-ray diffraction analysis.

### High-pressure electrical conductivity response

**Figure 6:**
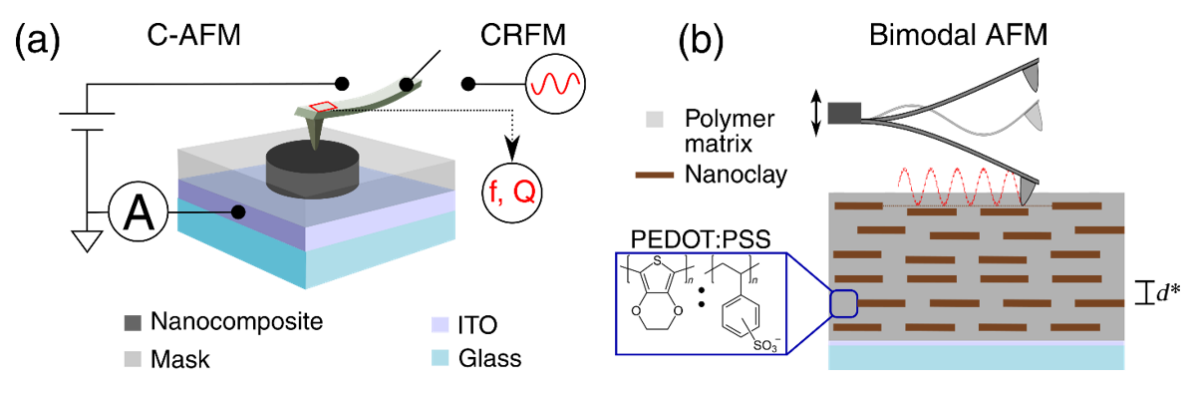
(a) Schematic illustration of the overall sample structure and the consecutive imaging of C-AFM and CRFM. (b) Illustration of the bimodal AFM setup and detailed nanocomposite description, including the expected sample response from the high-pressure treatment (red sinusoidal line).

The idealized structure of the coating is shown schematically in Figure 6b. For normal electrical transport, the presence of the nanoclay acts as a rectangular potential barrier with thickness of 1 nm, assuming that no other conduction paths exist. This is of course an idealization, but it offers useful physical insight. Since the nanocomposite contains several layers of nanoclay, a 1D rectangular multibarrier model [68] can be used to calculate the transmission probability of the electrons across the nanocomposite, where the wells correspond to the conductive polymer layers sandwiched between the nanoclay barriers. The effect of the high-pressure is considered by assuming a reduction in the distance between the barriers, which remains after the high-pressure AFM treatment. This is reasonable since the composite is not expected to be perfectly elastic. For the concentration of nanoclay used, the strain was measured at approximately 2% (Figure 5a). The barriers are 1 nm thick (nanoclay) separated by 2 nm (see Figure 5b), and the coating is taken to be 100 nm thick. It is assumed that the strain is distributed equally among the number of potential wells (or barriers) considered (this is another idealization). The Perimeter Free Electron Orbital Theory (PFEO) is a simple and useful tool for describing the energy levels for small conjugated molecules, so it is adopted for this analysis. In this model, the delocalized π electrons are treated as free electrons traveling in a loop around the conjugated molecular perimeter [69]. In the case of PEDOT, the perimeter is taken to be 0.774 nm [70]. Using the multibarrier tunneling model in conjunction with the PFEO, we can now calculate the transmission probability for the electrons corresponding to the PFEO energy levels, for different numbers of barriers (*N*) and for different levels of nanocomposite compression (strain). Based on the calculated changes in the transmission probability, it is now possible to predict (within the assumptions made) the direction of the changes in conductivity through the nanocomposite, as measured with C-AFM.

**Figure 7:**
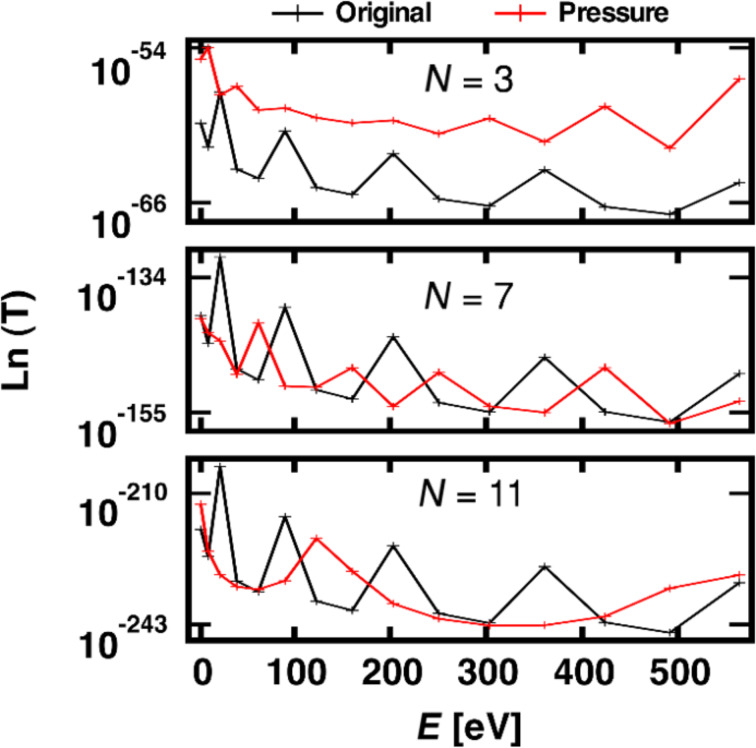
Transmission probability for a multibarrier system for the energy levels of the PCEO model. Three different numbers of barriers are shown (*N* = 3, 7 and 11) for the first 15 wave numbers (energy levels). “Original” (black curves) refers to the untreated coating, where the distance between barriers is 2 nm. “Pressure” (red curves) refers to the reduced inter-barrier distance following the high-pressure treatment.

From Figure 7 one can conclude that if only the few first layers (e.g., *N* = 3) of the nanocomposite experience strain under the application of pressure, the transmission probability should increase after the high-pressure treatment is performed. For the cases in which a larger number of well-barrier segments experiences strain (cases with larger *N* in Figure 7), the transmission probability shows a mixed response. Depending on the energy of the electrons, the transmission probability can increase or decrease following the high-pressure treatment.

The high-pressure treatment was performed using bimodal AFM to apply localized pressure to the nanocomposite. As discussed previously, the force applied by the tip to the surface increases with the use of higher eigenmodes and by increasing their free amplitude. Supporting Information File 1, Figure S5 shows the results of a virtual AFM numerical simulation, where the increase in peak forces as the free amplitude of the higher eigenmode increases is calculated using a quasi-3D model to represent the viscoelastic surface. Using the calculated peak force and the surface area of the spherical cap in contact with the sample, the pressure range varies from to 1.2 to 3.3 GPa. The overall experimental strategy is to measure the current before and after the bimodal AFM treatment, followed by measurement of the mechanical parameters. The consecutive imaging process and methods used are shown schematically in Figure 6.

**Figure 8:**
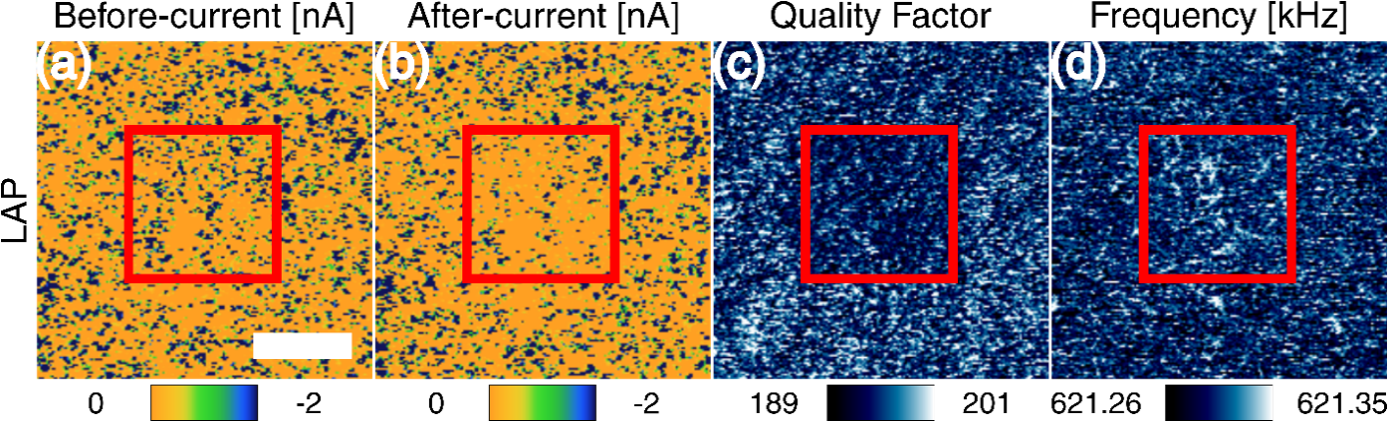
Electro-mechanical response of the transparent (thin) Laponite RD nanocomposite to the high-pressure treatment. Sequential imaging is used to acquire (a) current before the bimodal AFM treatment, (b) current after the treatment and, finally, (c,d) mechanical parameters after the treatment ((c) quality factor and (d) frequency), where the images have been automatically correlated. The bimodal-AFM-treated area is enclosed by the red square. The scale bar is 1 μm.

Figure 8 shows a summary of the results for LAP, including the current before and after the bimodal AFM treatment, and the mechanical parameters. The coating shows a local reduction of the out-of-plane current in the middle square (red square in Figure 8b). As predicted by the multibarrier model, the high-pressure results in an overall change in electron conductivity. The behavior of the current is not uniform, presumably due to the existence of a variety of alternate electron paths, where tunneling is not always the dominant mechanism. Clearly, the brick and mortar structure is not perfect and the concentration and distribution of platelets varies throughout and across the film, as shown by the distribution of conservative and dissipative interactions in bimodal AFM (Figure 4c,d). There are areas with higher concentration of polymer, where the current may remain unaffected after the bimodal AFM treatment. The reduction in current comes with a change in the mechanical parameters. Especially, the quality factor clearly shows a darker central square with dimensions close to 1.5 μm × 1.5 μm, which is the image size for the bimodal AFM treatment. The changes in the response of the treated area are attributed to small displacements of the nanoclay platelets. The displacements are expected to occur in the normal direction, with negligible rotation due to the flat platelet geometry (to confirm parallel compression of the nanoclay, the pixel density effect on the change of current is analyzed and shown in Supporting Information File 1, Figure S6). Very importantly, as shown in Figure 7, the multibarrier model predicts that for some values of the electron energy, the current can also increase. This was indeed observed experimentally, and Supporting Information File 1, Figure S7 shows an example of an increase in the current measured by C-AFM under the application of a larger bias voltage following the bimodal AFM treatment.

The MTM and PPSS thin samples were also investigated with the same procedure and under the same conditions as the thin LAP sample. Supporting Information File 1, Figure S8 shows the same set of results for these two samples. The bimodal AFM treatment in PPSS and MTM did not produce any observable change in the electro-mechanical response of the material. It is known that pressure can enhance the intermolecular interactions in π-conjugated polymers and influence their molecular geometry [71], thus modifying also their conductivity [72]. However, no changes were observed in PEDOT under the experimental conditions used. The lack of response for MTM was attributed to its larger average particle size of 260 nm [11], which is one order of magnitude larger than Laponite’s. Since the platelet area scales with the square of the particle size, the local pressure underneath the MTM platelet is reduced by two orders of magnitude, compared to Laponite, for a given value of the force applied by the AFM tip. Besides the platelet size difference, in MTM the current is segregated into “hotspots” and the formation of the skin layer also affects the surface structure, so the lack of response is not surprising.

In terms of applications, sensing devices based on nanoclays or layered structures have mainly relied on the effect of external stimuli that affect the organic layer. Typically, these assemblies swell in response to changes in temperature, pH, or the concentration of specific ions. In this work we have studied an invasive stimulus (high-pressure treatment) for nacre-inspired functional nanocomposites, which has an effect on the internal distribution of the nanoclay, which in turn affects the local conduction of electrons. Although the conductivity response is not uniform and its direction is not predictable a priori, the existence of a localized pressure response in conductive and transparent Laponite RD coatings could be useful with regards to the design of “smart” integrated systems, especially considering the other unique properties of the film, namely low gas permeability, fire retardancy, and excellent mechanical properties and flexibility.

## Conclusion

We have designed an optically transparent and electron conductive nanocomposite coating based on the polymer PEDOT:PSS and the nanoclays Laponite RD and Cloisite Na^+^, which exhibits a brick and mortar structure. We have thoroughly characterized the material in terms of transparency, local conductivity, nanomechanical properties and internal structure, and have also studied its response to the application of localized pressure with an AFM probe, whereby changes in conductivity were observed and explained in the context of a 1-dimensional multibarrier electron potential. Our observations on the conductivity-pressure relationship open up a new spectrum of applications for this type of multifunctional material, in light of its other features, including low gas permeability, fire retardancy, and excellent mechanical properties and flexibility. With regards to methodology, the comprehensive experimental approach followed, supplemented with numerical simulation, illustrates the systematic combination of intrinsic and complementary advantages of different AFM methods (C-AFM, CRFM and bimodal AFM) to modify, characterize and establish structure-functionality relations for advanced functional materials.

### Methods

#### Sample preparation

Nanoclay dispersions with 0.5 wt % of Laponite RD (LAP) or Cloisite Na^+^ (montmorillonite, MTM), both from Rockwood Industries, were prepared in deionized water (DI-H_2_O) and stirred for 24 h at 1500 RPM. The dispersions were decanted for a few days to remove large particles. The thickness of individual nanoclays was found to be approximately 1 nm, as shown in Supporting Information File 1, Figures S9 and S10. Poly(3,4-ethylenedioxythiophene)-poly(styrenesulfonate) (PEDOT:PSS, Sigma-Aldrich 768642, abbreviated as PPSS), a high-conductivity grade polymer, in 1.0 wt % solution in water was used as received. The core/shell nanoplatelets were prepared by slowly adding the nanoclay dispersion to a stirred polymer solution with a 33:67 (clay/polymer) weight ratio, which was further stirred for one hour. The dispersions were degassed in a sonicator. All samples were drop casted in a 3/8 inch circular mask with 80 μL of the dispersion and dried at 25 °C for 12 h. The dispersions with higher solids concentration (as prepared) were heated to 50 °C in order to ensure full solvent evaporation. The core/shell nanoplatelet dispersions were mixed with DI-H_2_O in 1:2, 1:4, 1:8 and 1:16 dilutions and casted. This reduces the amount of core/shell nanoplatelets per casting and results in thinner films. Three different substrates were used: silicon wafers (thickness measurements), indium tin oxide (ITO, electromechanical characterization) and glass slides (light spectroscopy). Silicon wafers (Ted Pella, Inc.) were sonicated in a sequence of isopropyl alcohol, ethanol, and DI-H_2_O; then heated with a butane torch (until bright orange glowing) for 30 s for cleaning. The sample thickness was measured with AFM by the scratch method (a sharp knife was used to remove the coating and expose the substrate). The ITO coated PET (Sigma-Aldrich 639281) with surface resistivity of 100 Ω/sq and the glass slides (micro cover glasses, 22 × 30 mm × 0.13–0.16 mm thick, Ted Pella, Inc.) were cleaned similarly (without torch). For convenience, the samples casted from the undiluted dispersion are referred to throughout the paper as “thick” and the samples casted from 1:16 dilution are referred to as “thin”.

#### Characterization techniques

An Asylum Research MFP-3D atomic force microscope equipped with an ARC2 SPM controller was used for all the scanning probe measurements. The operation of C-AFM required a modification of the system. An externally connected low-noise current amplifier (FEMTO^®^, DLPCA-200) was used to measure the current flowing between the conductive tip and the sample. The bias voltage was applied while the scanning conductive tip served as a movable nanoelectrode in continuous contact with the sample (Figure 6a). Bimodal AFM was used in the so-called amplitude modulated-open loop (AM-OL) scheme (shown in Figure 6b). In this scheme, the cantilever is excited at two eigenfrequencies simultaneously. The fundamental eigenmode is operated in amplitude modulation, i.e., there is a feedback loop modulating the oscillation amplitude for acquiring the topography of the sample, while the higher eigenmode (in this case the second eigenmode) is operated with constant excitation frequency and amplitude, without feedback [61].

CRFM-DART was used as implemented in the Asylum Research software. In general, the cantilever is shaken sinusoidally while in continuous contact with the sample, measuring two parameters: the resonance frequency and quality factor of the tip–sample junction (as shown in Figure 6a). Using the Euler–Bernoulli beam model interacting with a Kelvin–Voigt spring-dashpot element at the tip–sample junction, decoupling of the conservative and dissipative interactions of the tip–sample junction is possible [73]. In CRFM-DART, the amplitude and phase of the cantilever response are monitored at two frequencies, one lower and one higher than the contact-resonance frequency. Besides measuring the topography (by maintaining a constant deflection setpoint), the recorded amplitude and phase for each frequency are used to calculate the contact-resonance frequency and quality factor at each pixel [74]. “CRFM” refers to CRFM-DART throughout the paper.

The cantilevers used for the experiments were: Budget Sensors ContE-G and Multi75E-G, both Cr/Pt coated. Specific experimental parameters are given for the corresponding figures in Supporting Information File 1, pages S14 and S15. Amplitude versus distance or deflection versus distance curves were used to calibrate the amplitude of the first eigenmode or deflection in nanometers, respectively. The theoretical optical sensitivity (see Tab. 1 in [75]) was used to estimate the free amplitude of the second mode. The thermal noise method was used to calibrate the force constant of the cantilevers. CRFM and C-AFM measurements were carried out with a scan rate of 0.5 Hz over 256 × 256 pixels per image. Bimodal AFM images were obtained with a scan rate of 2 Hz over 256 × 256 pixels per image. All measurements were performed in ambient conditions (≈20 °C and ≈40% RH).

The scanning electron microscopy (SEM) images were taken using a FEI Teneo LV. Samples were casted on a glass slide, then cooled down with liquid nitrogen and broken in half. They were then placed at a 45-degree angle with respect to the electron gun, in order to observe the cross section. Optical transmittance spectroscopy was performed by using a CCS200 spectrometer connected to a SLS201 light source and a 2-inch integrating sphere (model IS200-4) procured from Thor Labs.

## Supporting Information

File 1 Additional experimental parameters and results.
