# Supplementary material for: High-stress study of bioinspired multifunctional PEDOT:PSS/nanoclay nanocomposites using AFM, SEM and numerical simulation
Source: Beilstein J Nanotechnol. 2017 Oct 4;8:2069–82. doi: 10.3762/bjnano.8.207 (PMC5647735; doi:10.3762/bjnano.8.207)
Supplement: File 1 — Additional experimental parameters and results. [file Beilstein_J_Nanotechnol-08-2069-s001.pdf]

Supporting Information

for

High-stress study of bioinspired multi-functional PEDOT:PSS/nanoclay nanocomposites using AFM, SEM and numerical simulation

Alfredo J. Diaz, Hanaul Noh, Tobias Meier and Santiago D. Solares[*,§]

Address: Department of Mechanical and Aerospace Engineering, The George Washington University, Washington, DC 20052, United States

Email: Santiago D. Solares - ssolares@gwu.edu

[*]Corresponding author

[§]Phone: +1 (202) 994-0372

Additional experimental parameters and results

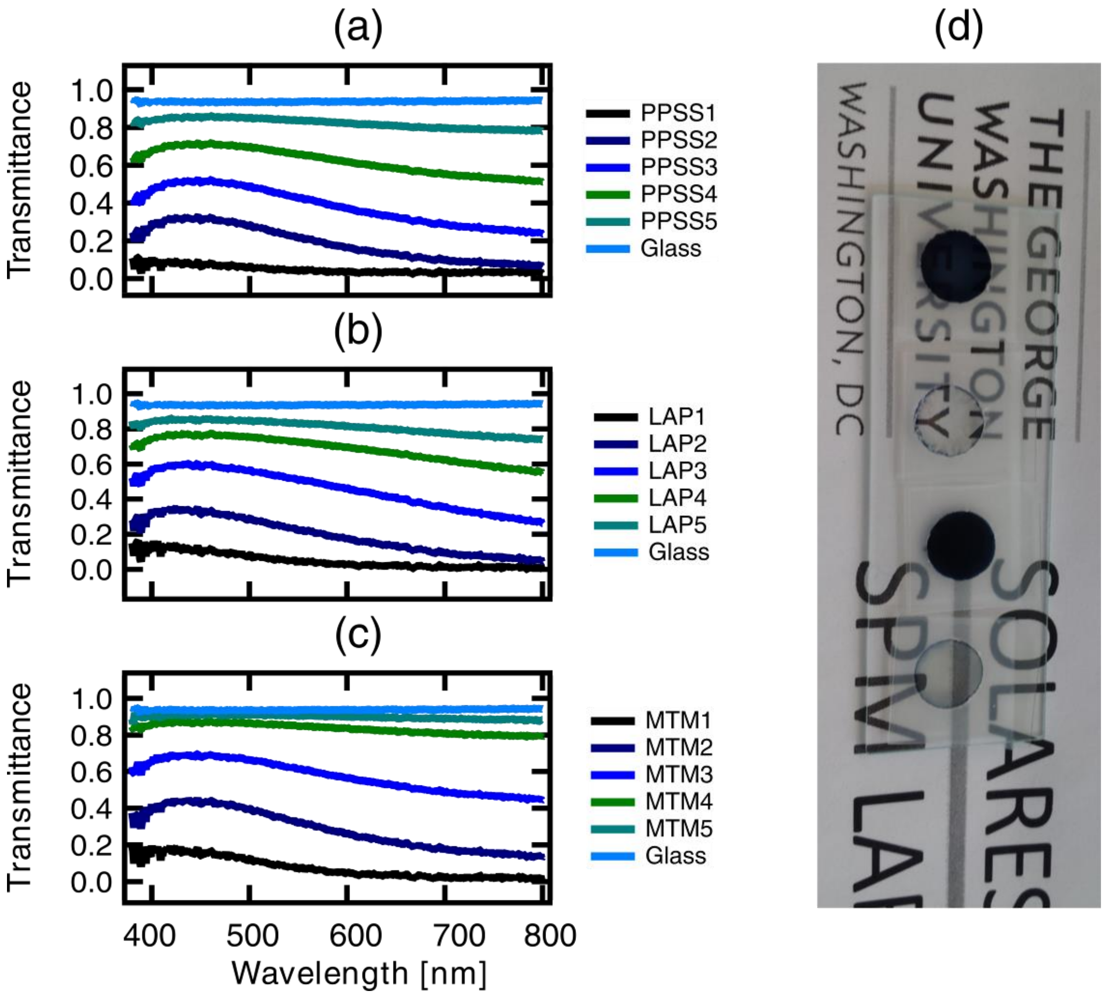

Figure S1: *Optical transmittance spectroscopy*. The full spectrum is shown in these figures for (a) PPSS, (b) LAP and (c) MTM. Samples identified as 1 are thickest and those identified as 5 are thinnest. Also, a visual comparison of the thick and thin samples is given. In (d), the top two samples are LAP and the bottom two samples are MTM. Analyzing the full spectra (a-c) for all the samples, it can be seen that the transmission is maximum on the blue end (around 475 nm) of the spectrum, and then decreases when moving into the red, which is the typical behavior measured for bare PEDOT:PSS films [1].

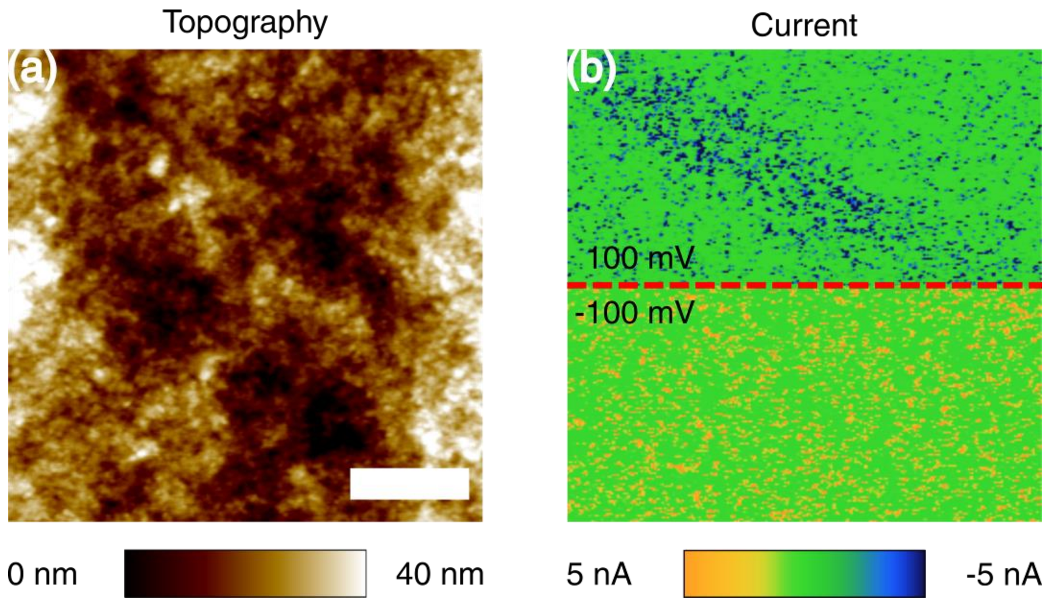

Figure S2: B*ias polarity dependance*. C-AFM was performed and the bias polarity was changed in the middle of the image. Ohmic behavior is observed in (b). The conductive spots switch from -5 nA (top image, blue spots) to +5 nA (bottom image, yellow spots) when the polarity is changed while maintaining a background of zero current (green). The scale bar is 1 μm.

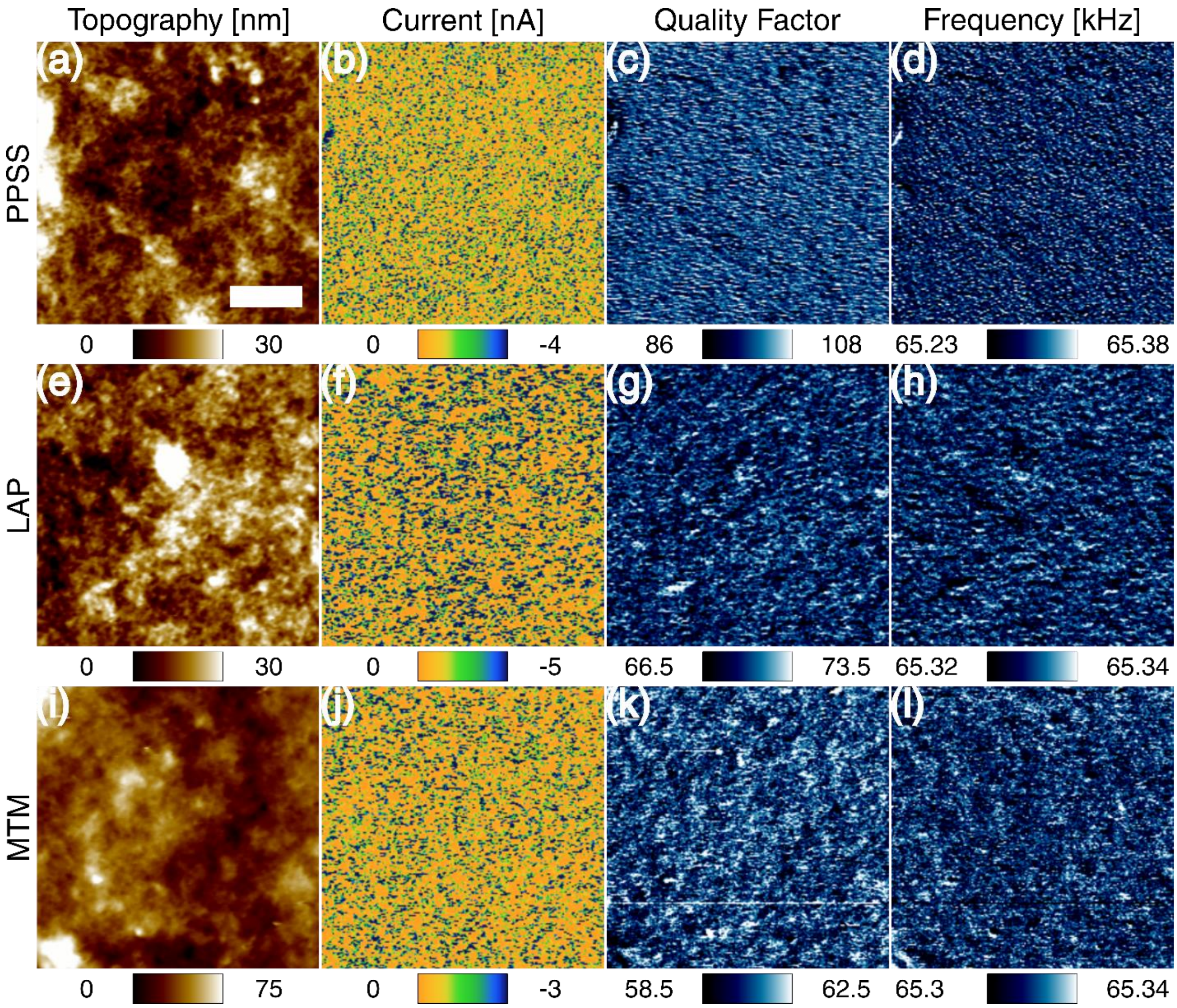

Figure S3: *Typical correlated electro-mechanical properties of the thick sam*ples: PEDOT:PSS (a-d), Laponite RD nanocomposite (e-h) and Cloisite Na^+ nanocomposite (i-l). The columns represent topography, current, quality factor and contact-resonance frequency, respectively, from left to right. The normal force applied to the surface during imaging was 3.6 nN (cantilever: ContE-G Cr/Pt coated). The free resonance frequency of the cantilever was 17.3 kHz. The scale bar is 1 μm. The distribution of electro-mechanical properties for all samples is similar.

Table S1: Parameters used for the strain calculation.

Parameters		Reference
E_m (Pa)	1.02E+09 (for 80nm thick film)	[2]
E_f (Pa)	1.78E+11	[3]
α (t/D)	0.04	[4]
σ_y (Pa)	4.28E+07 (casted film)	[5]
δ_m (g-cm^{-3})	1.011 (dried film)	[6]
δ_f (g-cm^{-3})	2.65	[4]
a	1.01	[7] For a similar system composed of PET/NaMMT
k	-9.52	
a_1	0.99	
k_1	-1.44	
B_1	4.52	
f - filler, m-matrix, E- Young's modulus, σ- yield strength, δ- density, t- thickness, D- diameter		

Comparison of different stress-strain models:

To verify the selection of the modified Pukánszky model for yield strength and the Hui-Shia model for the Young's modulus, the strain was calculated using several combinations of different models. Specifically, three models for yield strength and three models for Young's modulus are used in combination, resulting in 9 possibilities, whose results were compared with the experimental data. The models used [7] for yield strength are:

$$\sigma_c = \sigma_m(a - k\phi_f) \qquad \text{(S1)}$$

$$\sigma_c = \sigma_m\left(a_1 - k_1\phi_f^{0.5}\right) \quad \text{(S2)}$$

$$\sigma_c = \sigma_m\left(\frac{1 - \phi_f^{0.5}}{1 + 2.5\phi_f^{0.5}}\right)e^{B_1\phi_f^{0.5}} \quad \text{(S3)}$$

where c stands for composite, m for matrix, and f for filler (nanoclay); ϕ is the filler volume fraction, a and a_1 are fitting parameters close to 1; k and k_1 are constants that depend on clay thickness, and B_1 is a parameter that depends on the interfacial interactions. Equation (S1) defines a linear relationship between the yield strength of the nanocomposite and the volume fraction, while equation (S2) is a power law. The k's are parameters that only depend on the geometry of the nanoclay. The expressions are derived from the work of Nicolais and Narkis [8], which only considers the decrease of the effective load bearing cross-section [9]. In cases where these equations fitted the experimental results properly, the k's are considered to be a function of clay thickness, intercalation/exfoliation level and interfacial properties. On the other hand, equation (S3), referred to as the modified Pukánszky model [3], combines: (i) a term that indicates the decrease of effective load-bearing cross section due to filler introduction, similar to Nicolais' and Narkis' model $\left(\frac{1 - \phi_f^{0.5}}{1 + 2.5\phi_f^{0.5}}\right)$, (ii) a B_1 that is determined by the polymer/nanoclay interfacial properties, and (iii) the nonlinear relation observed in polymer/nanoclay composites between the yield strength and the filler volume fraction. The latter can be considered as the most comprehensive model.

For the Young's modulus, the three models used are the Voigt [10], Halpin-Tsai [11] and Hui-Shia [12] models (equations S4 to S6 respectively). These models have been widely applied to study the properties of polymer/nanoclay composites. The Voigt model is simply a weighted mean of the properties of the individual components. The Halpin-Tsai model is a semi-empirical relation that was originally developed for fiber-like inclusions in polymers. Hui-Shia derived

equations for predicting the Young's modulus of composites with aligned reinforcements (with emphasis on plate-like reinforcements), whereby good agreement of the theoretical predictions with experimental results was found. The strain behavior for all the combinations is shown in Figure S4. From visual inspection, two curves (SQRT-Hui-Shia and Pukánszky-Hui-Shia) are similar in terms of shape and magnitude to the experimental results obtained. The experimental results are well fitted by an exponential function, which showed the best fit for exponential behavior for the Pukánszky-Hui-Shia model (R^2 = 0.99).

$$E_c = \phi_m E_m + \phi_f E_f \qquad \text{(S4)}$$

$$E_c = E_m \left[\frac{1 + 2\eta\phi_f}{1 - \eta\phi_f}\right]; \quad \eta = \frac{\frac{E_f}{E_m} - 1}{\frac{E_f}{E_m} + 2} \qquad \text{(S5)}$$

$$E_c = \frac{E_m}{1 - \frac{\phi_f}{\xi}}\ ; \qquad \alpha = \frac{t}{D} = \frac{1}{25}$$

$$g = \left[\frac{\alpha}{(1-\alpha^2)^{1.5}}\right]\left[-\alpha\sqrt{1-\alpha} + \cos^{-1}\alpha\right] \qquad \text{(S6)}$$

$$\xi = \phi_f + \frac{E_m}{E_f - E_m} + 3(1-\phi_f)\left[\frac{(1-g)\alpha^2 - \frac{g}{2}}{\alpha^2 - 1}\right]$$

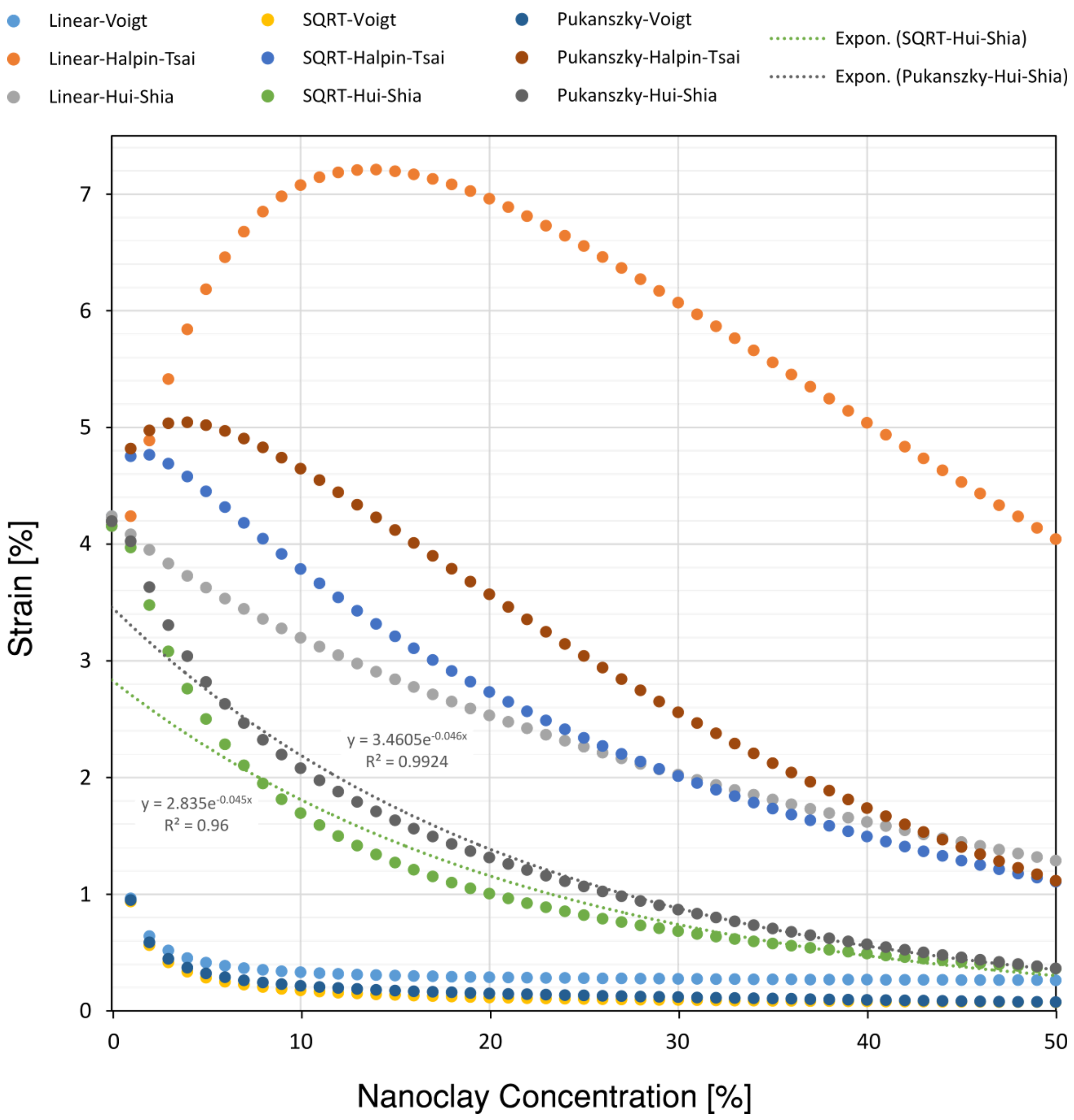

Figure S4: *Strain prediction*, using several models described in the text, for PEDOT:PSS and the nanoclay composites. The designation "linear" corresponds to equation (S1). SQRT corresponds to equation (S2). The parameters used are given in Table S1.

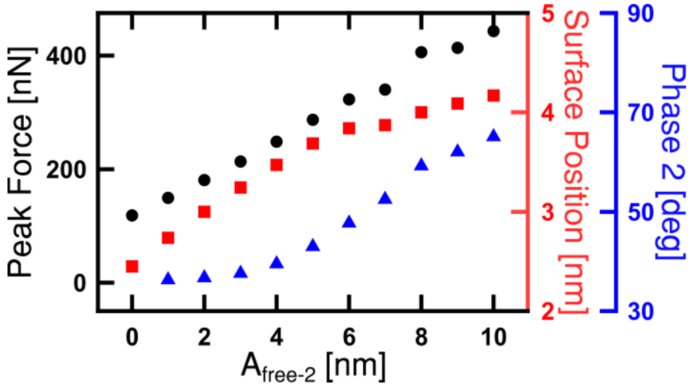

Figure S5: *Virtual AFM simulation of peak forces, change in surface position (indentation) and phase of the second eigenmode for a cantilever operating in bimodal AFM interacting with a quasi-3D standard linear solid surface* [13] *for different free oscillation amplitudes.* The parameters for the surface are: $k_1 = 7.5 \times 10^{-2}$ N/m/nm^2, $k_2 = 7.5 \times 10^{-2}$ N/m/nm^2, $c_{diss} = 1 \times 10^{-7}$ Ns/m/nm^2 and $k_{int} = 0.24 \times 10^{11}$ N/m^2. The software and details of the method are described elsewhere [13]. The first eigenmode of the cantilever has a free amplitude of 100 nm and a setpoint of 75%. The cantilever parameters are taken to be similar to the commercially available AC160TS probe used in this study (k_1 = 23 N/m, f_1 = 380 kHz). As the free amplitude of the higher mode increases, peak forces, change in surface position and the average phase value also increase.

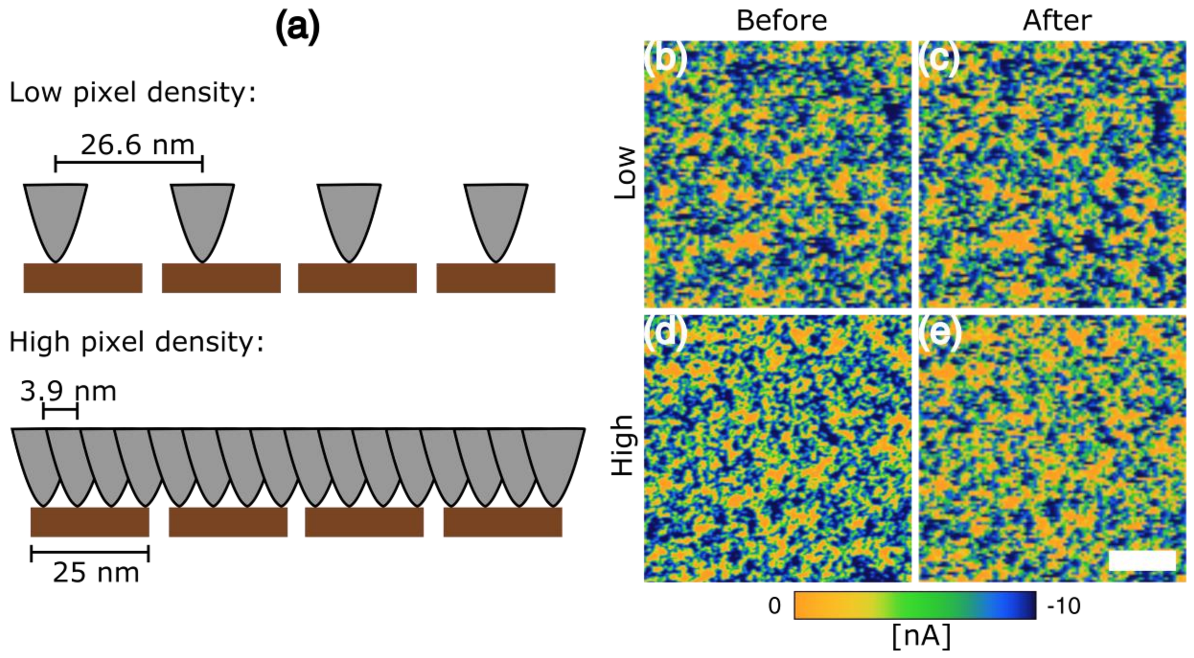

Figure S6: *Effect of pixel density.* (a) Schematic for the effect of pixel density on the change in conductivity produced by the bimodal AFM treatment, based on the number of impacts between the tip and the nanoclays (the polymer surrounding the nanoclays is not depicted in the schematic). The quantity of points and lines (forming a grid) was increased from 75 (low density) to 512 (high density), thus producing a grid of equally spaced pixels for a 2 µm × 2 µm bimodal AFM image. Current images before (b, d) and after (c, e) the bimodal AFM treatment for low (b-c) and high (d-e) pixel density, respectively. The scale bar is 500 nm. In order to properly compare the measurements before and after the treatment, the current in the measured area is added. The results are summarized in Table S2. The sample treated with low pixel density showed a decrease of 2.14% in the current, while the high-pixel-density bimodal AFM treatment showed a reduction of 13.28%. The larger reduction in current is directly related to the quantity of pixels, thus the tip squeezes nanoclays closer to each other when more tip-sample impacts take place for a given region.

Table S2: Values for the addition of the current pixels shown in Figure S6 (b-e) and the percentage difference before and after the bimodal AFM treatment.

Pixel density	Addition of pixels [A]		Percentage Difference [%]
low	before	6.26E-05	2.14
	after	6.12E-05	
high	before	6.23E-05	13.28
	after	5.45E-05	

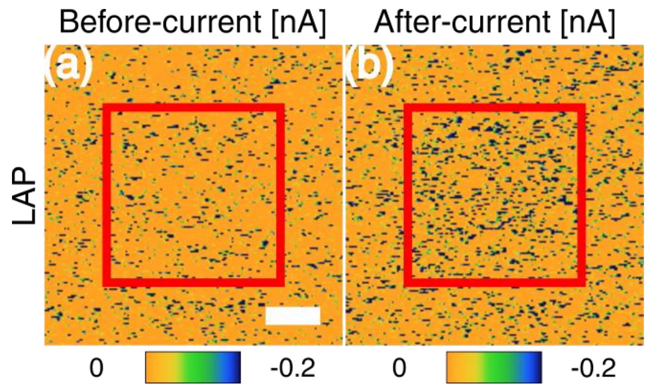

Figure S7: Electrical response of the transparent (thin) Laponite RD nanocomposite (same concentration as the high-pressure experiments reported in the main text) to the high-pressure treatment. Sequential imaging is used to acquire (a) current before the bimodal AFM treatment and (b) current after the treatment. A Multi75E-G Cr/Pt coated cantilever was used. The bimodal-AFM-treated area is enclosed by the red square. In this case the bias voltage is 500 mV. All the previous characterization with C-AFM was performed at a bias voltage of 100 mV. The scale bar is 1 μm. The local current after the bimodal AFM treatment shows an increase.

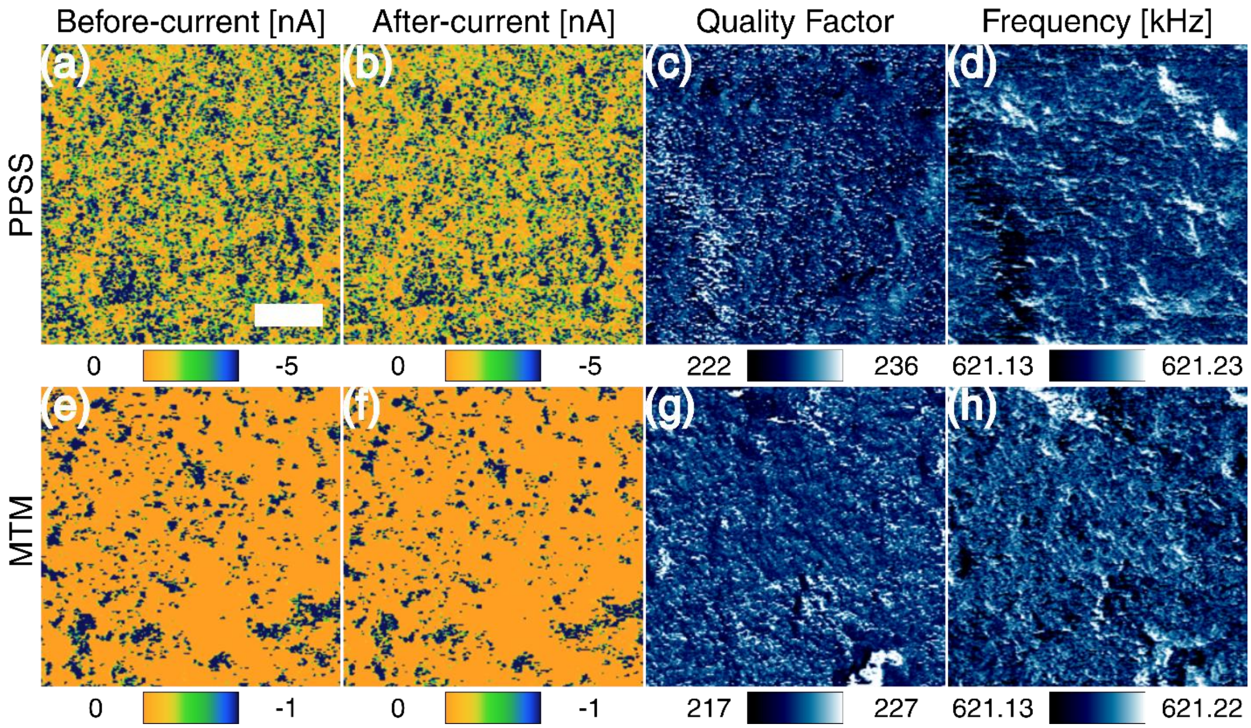

Figure S8: Attempts at subsurface modification for PPSS and MTM via by the same methodology and parameters used for the experiments shown in Figure 8. There is no measurable response for the PPSS and MTM films. The scale bar is 1 μm.

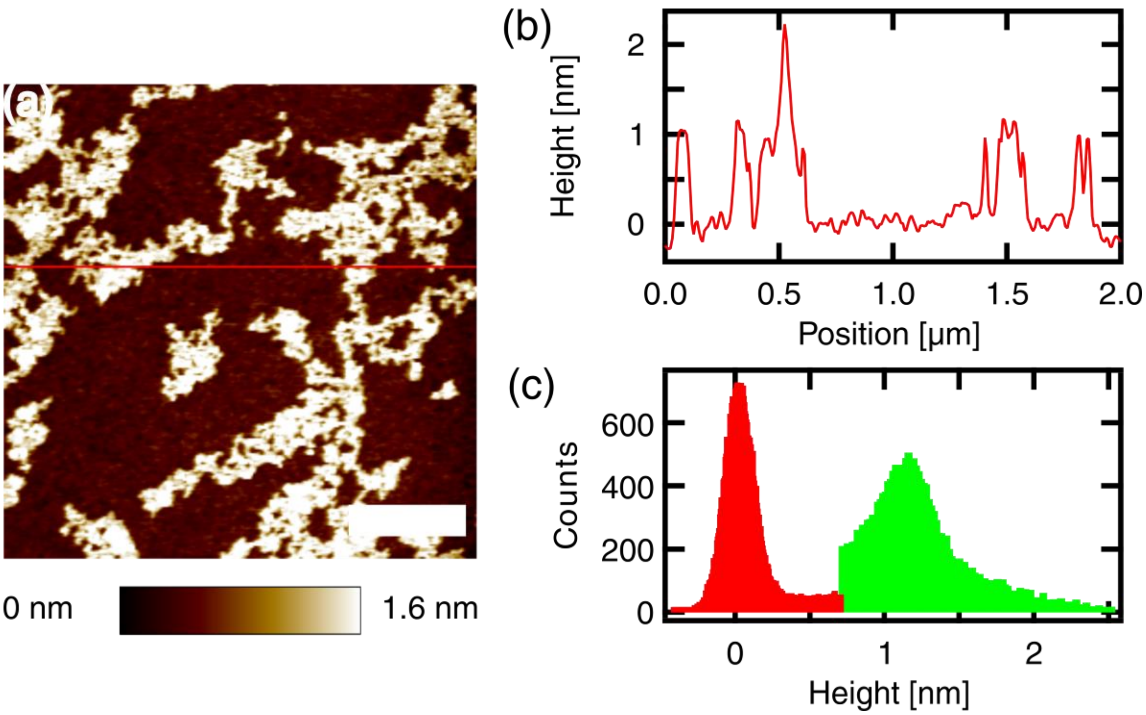

Figure S9: *Laponite RD morphology*. Topography image taken in tapping mode AFM (a). The cross section (b) shows the typical 1 nm thick platelets. The statistical analysis (c) also shows larger thicknesses, since as shown in the cross section, there are some double layers. The 0.5 wt. % solution was diluted to 1:20 volume and spin coated for 30 s @ 1000 RPM on a cleaned silicon substrate. The scale bar is 500 nm.

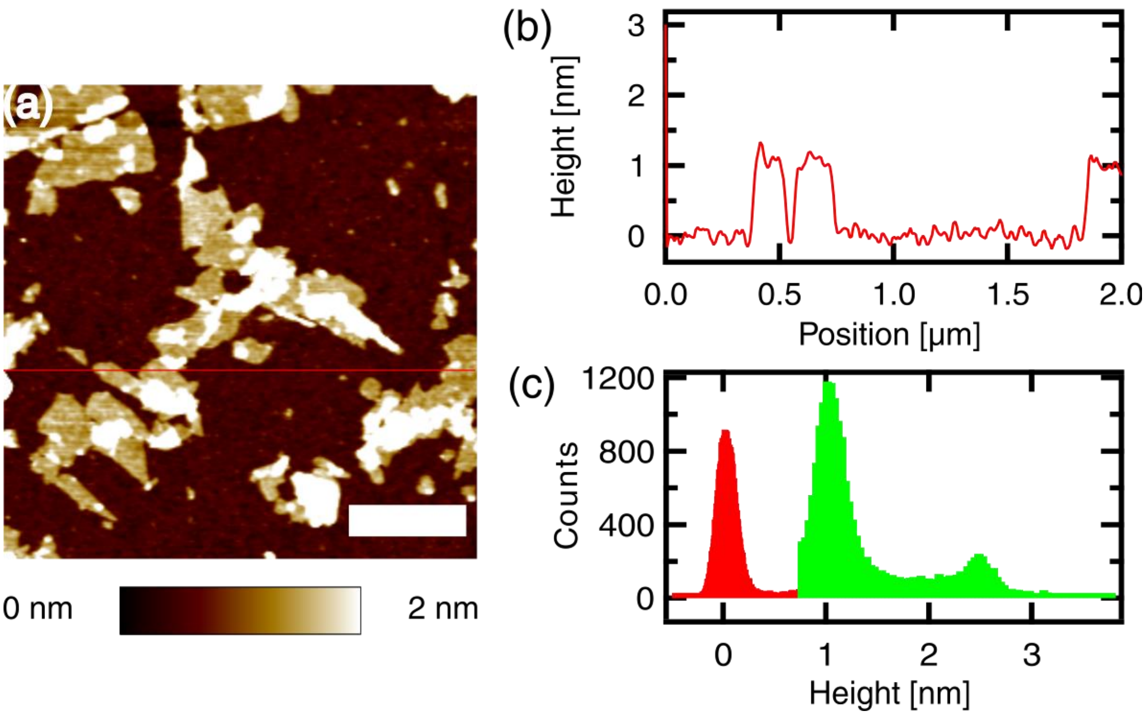

Figure S10: *Cloisite* Na^+ *morphology*. Similar information as in Figure S8. The 0.5 wt. % solution was diluted to 1:20 volume and 3 µL were drop casted onto a cleaned silicon substrate. The scale bar is 500 nm.

Experimental parameters for the figures in the main text:

Figure 1: *The thick samples were imaged with 3.6 nN of normal force and the thin samples with 2.6 nN, both using a ContE-G Cr/Pt coated cantilever.*

Figure 2: *All images (5 µm × 5 µm) were taken with the same cantilever (ContE-G Cr/Pt coated) using a normal force of 2.4 nN. The free resonance frequency of the cantilever was 15 kHz.*

Figure 3: *The normal force applied to the surface during scanning is 2.4 nN (cantilever: ContE-G Cr/Pt coated). The free resonance frequency of the cantilever was 14.1 kHz.*

Figure 4: *The free amplitude of the first eigenmode was 72 nm with a setpoint of 80%, using an AC160TS cantilever with a fundamental force constant of 23.7 N/m. The amplitude of the second eigenmode was varied in order to optimize the contrast in the energy quantities.*

Figure 5: (a) *For the experiments, the free amplitude of the first eigenmode was 74 nm with a setpoint of 67% using an AC160TS cantilever with a fundamental force constant of 23 N/m. Three different amplitudes (2, 10 and 20 nm) were used for the second eigenmode, as shown in the legend. The samples were dried at 50 ºC in a 60% RH environment to produce a high-quality transparent coating for all nanoclay concentrations. All the AFM measurements were performed at a relative humidity of 25%. (b) Two separate experiments were performed in repulsive regime AM-AFM with a free amplitude of 60 and 120 nm, respectively, and a setpoint of 50%.*

Figure 8: *A Multi75E-G Cr/Pt coated cantilever was used. The normal force applied was 12.5 nN for the C-AFM and CRFM measurements. The Bimodal AFM treatment was performed using the same cantilever with the following parameters:* f_1 *= 68.6 kHz,* A_{free-1} *= 155 nm,* k_1 *= 4.11 N/m, setpoint = 50 %,* f_2 *= 440 kHz and* A_{free2} *= 45 nm (estimated using the theoretical optical sensitivity). Through the relationship* $k_i = k_1(f_i/f_1)^2$*, the higher (second) mode force constant can be approximated as* k_2 *=168.1 N/m. Three consecutive bimodal AFM images (1.5* μ*m × 1.5* μ*m) were obtained, each taking approximately 2 minutes.*

References

1. McCarthy, J.; Hanley, C.; Brennan, L.; Lambertini, V.; Gun'ko, Y. *J. Mater. Chem. C* **2014**, *2*, 764–770.
2. Greco, F.; Zucca, A.; Taccola, S.; Menciassi, A.; Fujie, T.; Haniuda, H.; Takeoka, S.; Dario, P.; Mattoli, V. *Soft Matter* **2011**, *7*, 10642.
3. Zare, Y.; Garmabi, H. *Appl. Clay Sci.* **2015**, *105–106*, 66–70.
4. Liff, S. M.; Kumar, N.; McKinley, G. H. *Nat. Mater.* **2007**, *6*, 76–83.
5. Lang, U.; Naujoks, N.; Dual, J. *Synth. Met.* **2009**, *159*, 473–479.
6. Tevi, T.; Saint Birch, S. W.; Thomas, S. W.; Takshi, A. *Synth. Met.* **2014**, *191*, 59–65.
7. Zare, Y. *Compos. Part B Eng.* **2015**, *73*, 111–117.
8. Nicolais, L.; Narkis, M. *Polym. Eng. Sci.* **1971**, *11*, 194–199.
9. Turcsányi, B.; Pukánszky, B.; Tüdõs, F. *J. Mater. Sci. Lett.* **1988**, *7*, 160–162.
10. Voigt, W. *Ann. Phys.* **1889**, *274*, 573–587.
11. Halpin, J. C.; Kardos, J. L. *Polym. Eng. Sci.* **1976**, *16*, 344–352.
12. Hui, C. Y.; Shia, D. *Polym. Eng. Sci.* **1998**, *38*, 774–782.
13. Solares, S. D. *Beilstein J. Nanotechnol.* **2016**, *7*, 554–571.
